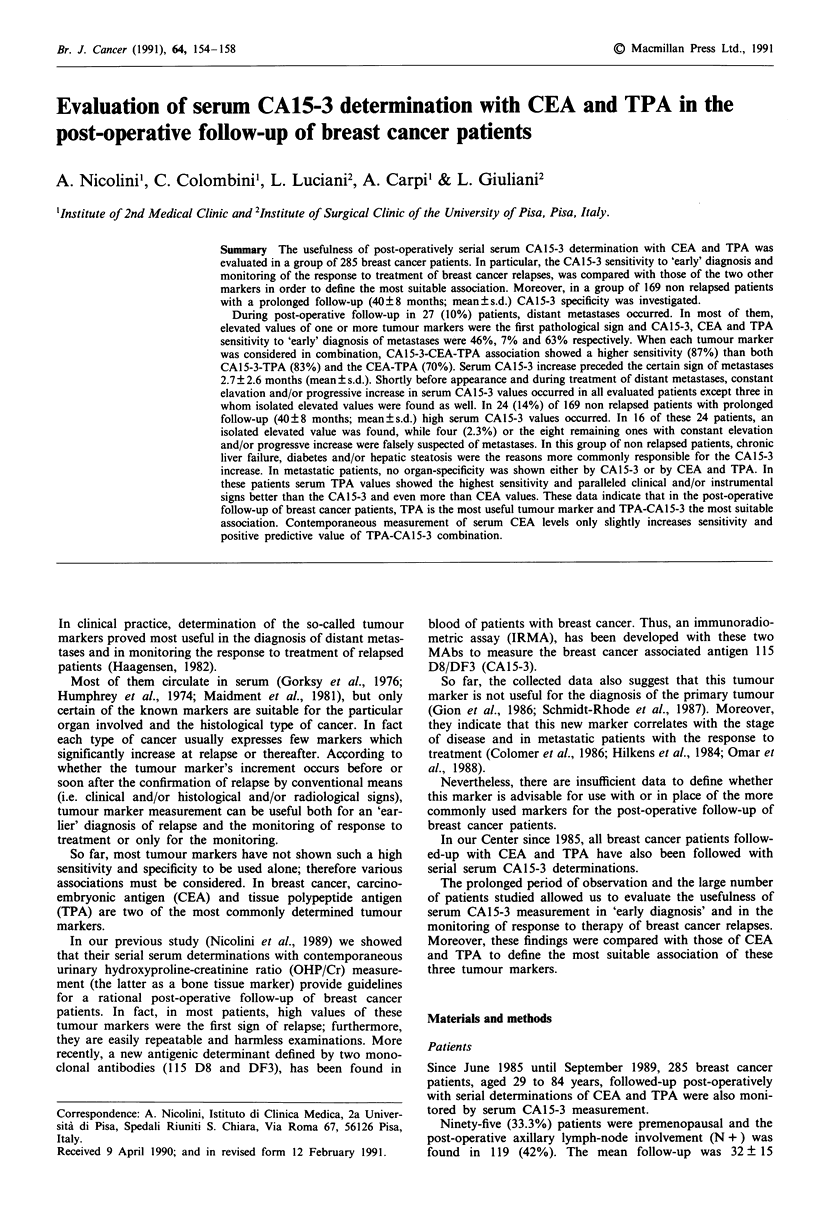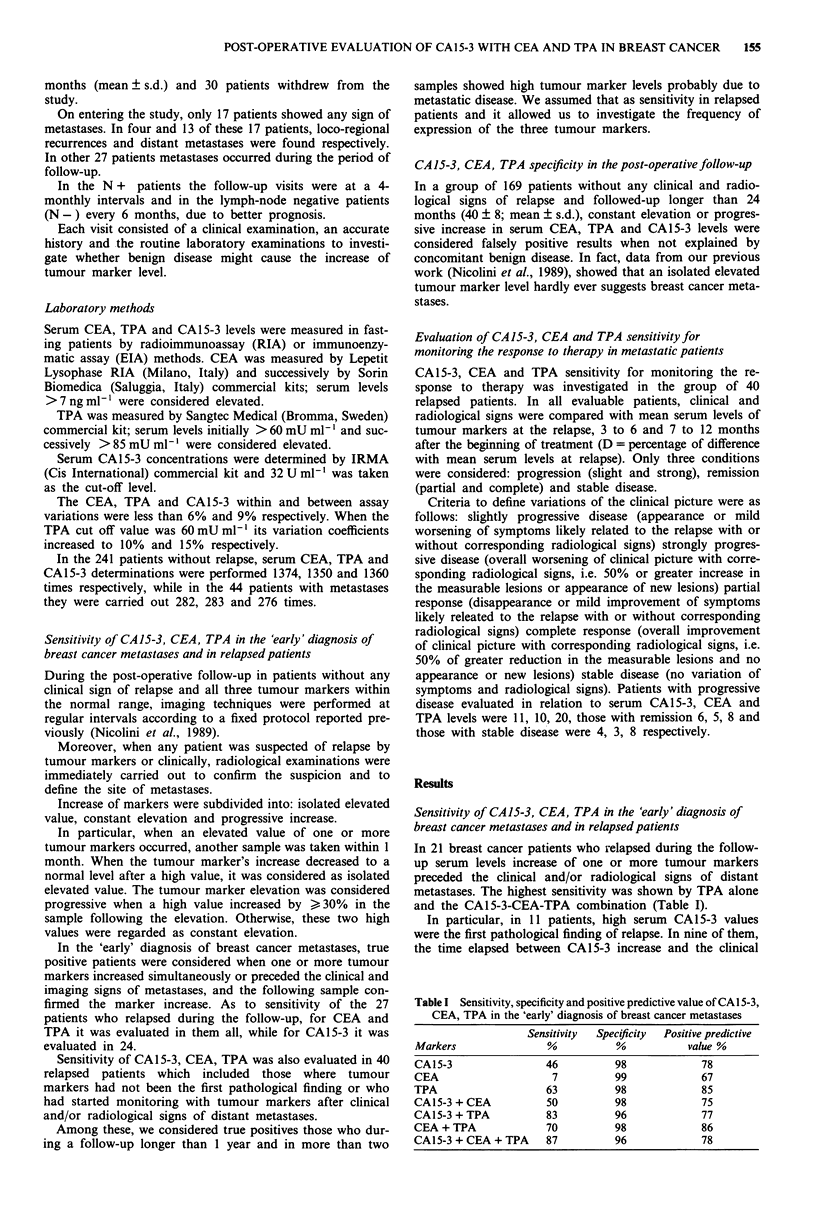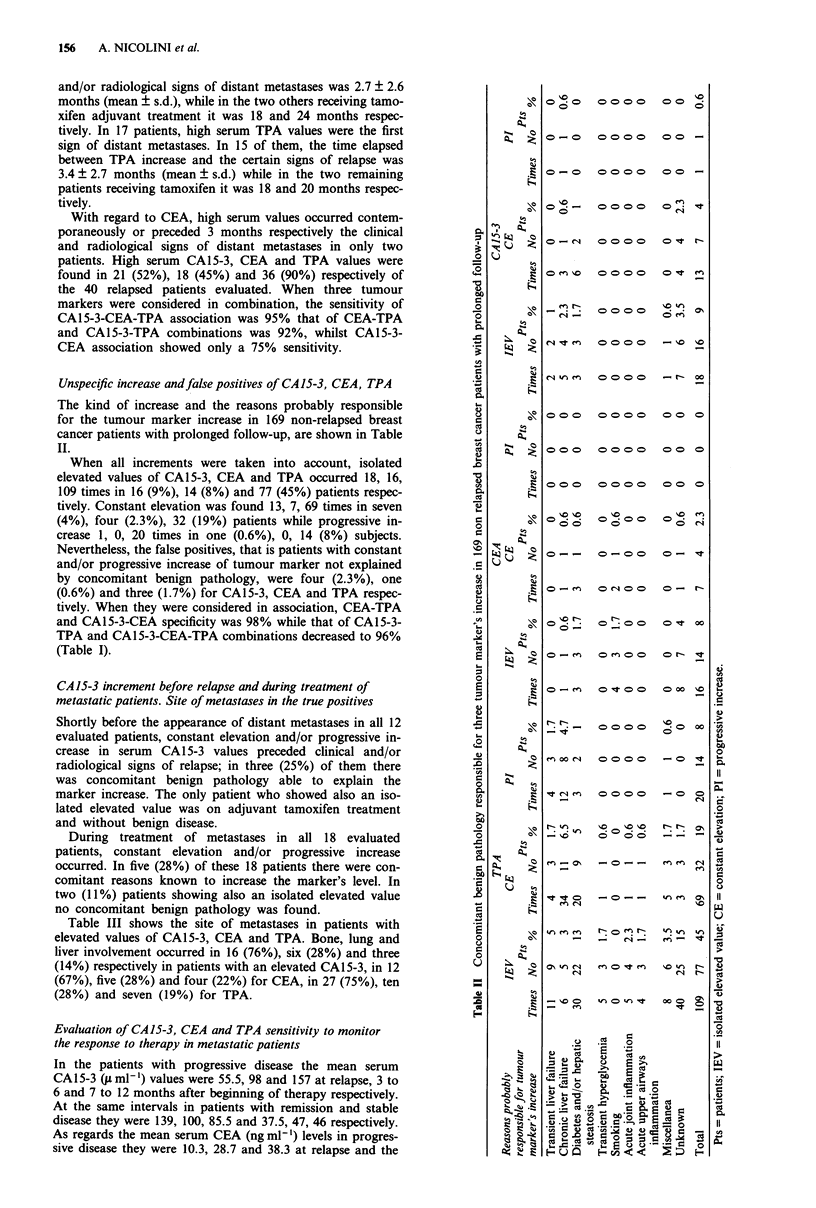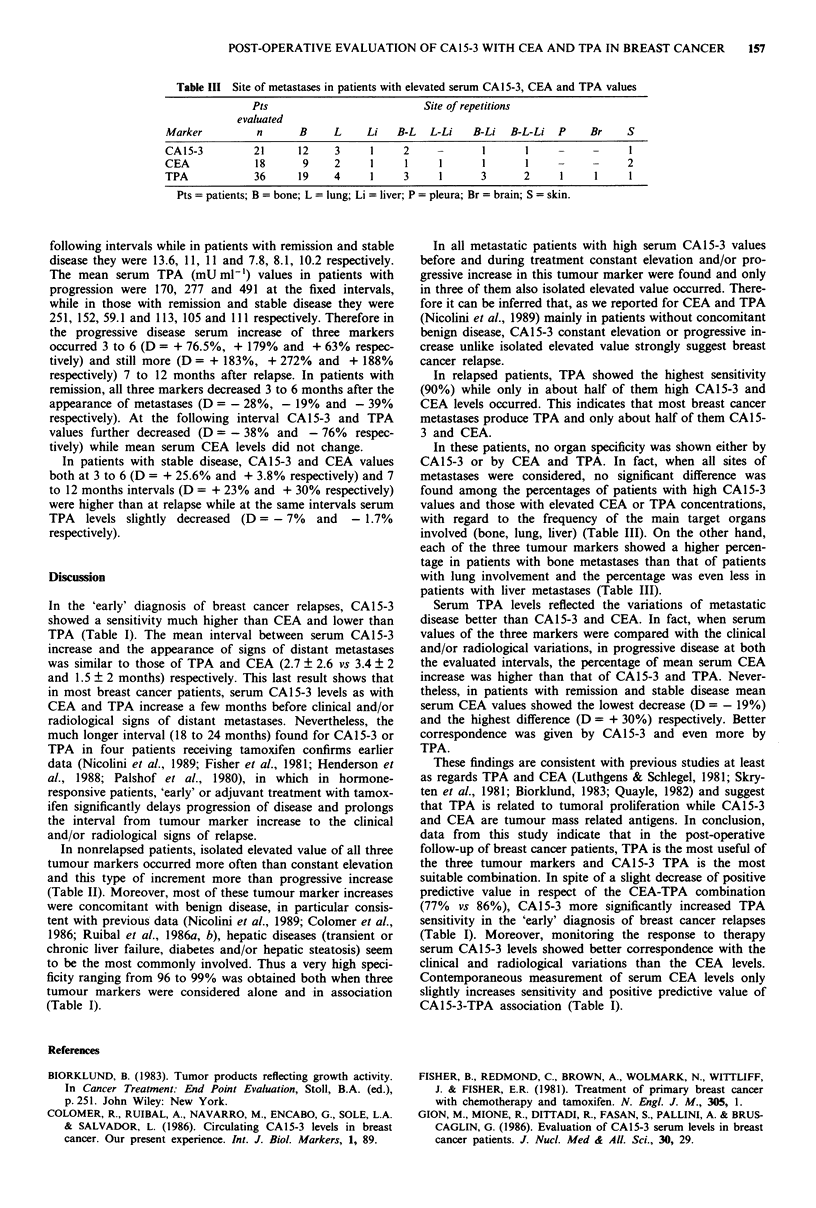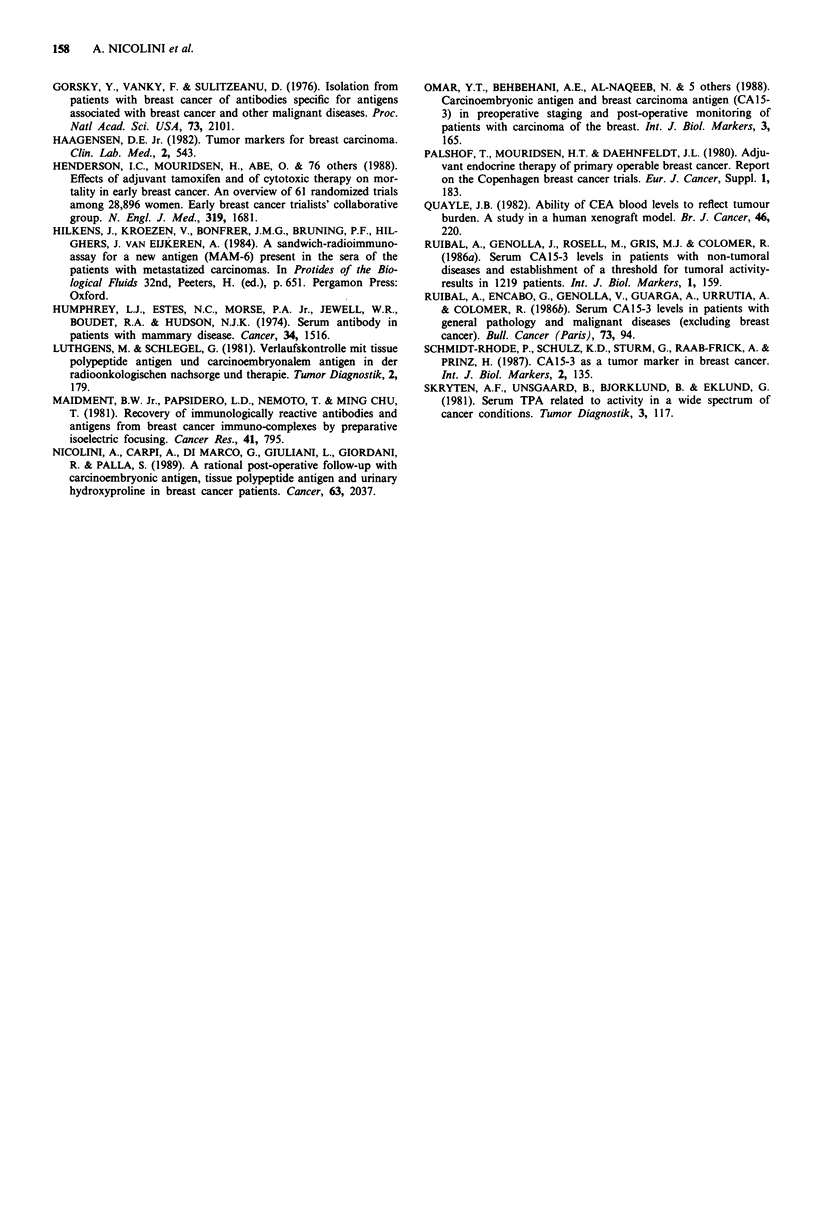# Evaluation of serum CA15-3 determination with CEA and TPA in the post-operative follow-up of breast cancer patients.

**DOI:** 10.1038/bjc.1991.260

**Published:** 1991-07

**Authors:** A. Nicolini, C. Colombini, L. Luciani, A. Carpi, L. Giuliani

**Affiliations:** Institute of 2nd Medical Clinic, University of Pisa, Italy.

## Abstract

The usefulness of post-operatively serial serum CA15-3 determination with CEA and TPA was evaluated in a group of 285 breast cancer patients. In particular, the CA15-3 sensitivity to 'early' diagnosis and monitoring of the response to treatment of breast cancer relapses, was compared with those of the two other markers in order to define the most suitable association. Moreover, in a group of 169 non relapsed patients with a prolonged follow-up (40 +/- 8 months; mean +/- s.d.) CA15-3 specificity was investigated. During post-operative follow-up in 27 (10%) patients, distant metastases occurred. In most of them, elevated values of one or more tumour markers were the first pathological sign and CA15-3, CEA and TPA sensitivity to 'early' diagnosis of metastases were 46%, 7% and 63% respectively. When each tumour marker was considered in combination, CA15-3-CEA-TPA association showed a higher sensitivity (87%) than both CA15-3-TPA (83%) and the CEA-TPA (70%). Serum CA15-3 increase preceded the certain sign of metastases 2.7 +/- 2.6 months (mean +/- s.d.). Shortly before appearance and during treatment of distant metastases, constant elevation and/or progressive increase in serum CA15-3 values occurred in all evaluated patients except three in whom isolated elevated values were found as well. In 24 (14%) of 169 non relapsed patients with prolonged follow-up (40 +/- 8 months; mean +/- s.d.) high serum CA15-3 values occurred. In 16 of these 24 patients, an isolated elevated value was found, while four (2.3%) or the eight remaining ones with constant elevation and/or progressive increase were falsely suspected of metastases. In this group of non relapsed patients, chronic liver failure, diabetes and/or hepatic steatosis were the reasons more commonly responsible for the CA15-3 increase. In metastatic patients, no organ-specificity was shown either by CA15-3 or by CEA and TPA. In these patients serum TPA values showed the highest sensitivity and paralleled clinical and/or instrumental signs better than the CA15-3 and even more than CEA values. These data indicate that in the post-operative follow-up of breast cancer patients, TPA is the most useful tumour marker and TPA-CA15-3 the most suitable association. Contemporaneous measurement of serum CEA levels only slightly increases sensitivity and positive predictive value of TPA-CA15-3 combination.


*Br. J. Cancer* (1991), **64**, 154–158 © Macmillan Press Ltd., 1991

### Evaluation of serum CA15-3 determination with CEA and TPA in the post-operative follow-up of breast cancer patients

A. Nicolini[1], C. Colombini[1], L. Luciani[2], A. Carpi[1] & L. Giuliani[2]

[1]*Institute of 2nd Medical Clinic and* [2]*Institute of Surgical Clinic of the University of Pisa, Pisa, Italy.*

**Summary** The usefulness of post-operatively serial serum CA15-3 determination with CEA and TPA was evaluated in a group of 285 breast cancer patients. In particular, the CA15-3 sensitivity to 'early' diagnosis and monitoring of the response to treatment of breast cancer relapses, was compared with those of the two other markers in order to define the most suitable association. Moreover, in a group of 169 non relapsed patients with a prolonged follow-up (40 ± 8 months; mean ± s.d.) CA15-3 specificity was investigated.

During post-operative follow-up in 27 (10%) patients, distant metastases occurred. In most of them, elevated values of one or more tumour markers were the first pathological sign and CA15-3, CEA and TPA sensitivity to 'early' diagnosis of metastases were 46%, 7% and 63% respectively. When each tumour marker was considered in combination, CA15-3-CEA-TPA association showed a higher sensitivity (87%) than both CA15-3-TPA (83%) and the CEA-TPA (70%). Serum CA15-3 increase preceded the certain sign of metastases 2.7 ± 2.6 months (mean ± s.d.). Shortly before appearance and during treatment of distant metastases, constant elavation and/or progressive increase in serum CA15-3 values occurred in all evaluated patients except three in whom isolated elevated values were found as well. In 24 (14%) of 169 non relapsed patients with prolonged follow-up (40 ± 8 months; mean ± s.d.) high serum CA15-3 values occurred. In 16 of these 24 patients, an isolated elevated value was found, while four (2.3%) or the eight remaining ones with constant elevation and/or progressve increase were falsely suspected of metastases. In this group of non relapsed patients, chronic liver failure, diabetes and/or hepatic steatosis were the reasons more commonly responsible for the CA15-3 increase. In metastatic patients, no organ-specificity was shown either by CA15-3 or by CEA and TPA. In these patients serum TPA values showed the highest sensitivity and paralleled clinical and/or instrumental signs better than the CA15-3 and even more than CEA values. These data indicate that in the post-operative follow-up of breast cancer patients, TPA is the most useful tumour marker and TPA-CA15-3 the most suitable association. Contemporaneous measurement of serum CEA levels only slightly increases sensitivity and positive predictive value of TPA-CA15-3 combination.

In clinical practice, determination of the so-called tumour markers proved most useful in the diagnosis of distant metastases and in monitoring the response to treatment of relapsed patients (Haagensen, 1982).

Most of them circulate in serum (Gorksy *et al.*, 1976; Humphrey *et al.*, 1974; Maidment *et al.*, 1981), but only certain of the known markers are suitable for the particular organ involved and the histological type of cancer. In fact each type of cancer usually expresses few markers which significantly increase at relapse or thereafter. According to whether the tumour marker's increment occurs before or soon after the confirmation of relapse by conventional means (i.e. clinical and/or histological and/or radiological signs), tumour marker measurement can be useful both for an 'earlier' diagnosis of relapse and the monitoring of response to treatment or only for the monitoring.

So far, most tumour markers have not shown such a high sensitivity and specificity to be used alone; therefore various associations must be considered. In breast cancer, carcinoembryonic antigen (CEA) and tissue polypeptide antigen (TPA) are two of the most commonly determined tumour markers.

In our previous study (Nicolini *et al.*, 1989) we showed that their serial serum determinations with contemporaneous urinary hydroxyproline-creatinine ratio (OHP/Cr) measurement (the latter as a bone tissue marker) provide guidelines for a rational post-operative follow-up of breast cancer patients. In fact, in most patients, high values of these tumour markers were the first sign of relapse; furthermore, they are easily repeatable and harmless examinations. More recently, a new antigenic determinant defined by two monoclonal antibodies (115 D8 and DF3), has been found in blood of patients with breast cancer. Thus, an immunoradiometric assay (IRMA), has been developed with these two MAbs to measure the breast cancer associated antigen 115 D8/DF3 (CA15-3).

So far, the collected data also suggest that this tumour marker is not useful for the diagnosis of the primary tumour (Gion *et al.*, 1986; Schmidt-Rhode *et al.*, 1987). Moreover, they indicate that this new marker correlates with the stage of disease and in metastatic patients with the response to treatment (Colomer *et al.*, 1986; Hilkens *et al.*, 1984; Omar *et al.*, 1988).

Nevertheless, there are insufficient data to define whether this marker is advisable for use with or in place of the more commonly used markers for the post-operative follow-up of breast cancer patients.

In our Center since 1985, all breast cancer patients followed-up with CEA and TPA have also been followed with serial serum CA15-3 determinations.

The prolonged period of observation and the large number of patients studied allowed us to evaluate the usefulness of serum CA15-3 measurement in 'early diagnosis' and in the monitoring of response to therapy of breast cancer relapses. Moreover, these findings were compared with those of CEA and TPA to define the most suitable association of these three tumour markers.

### Materials and methods

### *Patients*

Since June 1985 until September 1989, 285 breast cancer patients, aged 29 to 84 years, followed-up post-operatively with serial determinations of CEA and TPA were also monitored by serum CA15-3 measurement.

Ninety-five (33.3%) patients were premenopausal and the post-operative axillary lymph-node involvement (N + ) was found in 119 (42%). The mean follow-up was 32 ± 15

Correspondence: A. Nicolini, Istituto di Clinica Medica, 2a Università di Pisa, Spedali Riuniti S. Chiara, Via Roma 67, 56126 Pisa, Italy.
Received 9 April 1990; and in revised form 12 February 1991.

months (mean ± s.d.) and 30 patients withdrew from the study.

On entering the study, only 17 patients showed any sign of metastases. In four and 13 of these 17 patients, loco-regional recurrences and distant metastases were found respectively. In other 27 patients metastases occurred during the period of follow-up.

In the N + patients the follow-up visits were at a 4-monthly intervals and in the lymph-node negative patients (N – ) every 6 months, due to better prognosis.

Each visit consisted of a clinical examination, an accurate history and the routine laboratory examinations to investigate whether benign disease might cause the increase of tumour marker level.

*Laboratory methods*

Serum CEA, TPA and CA15-3 levels were measured in fasting patients by radioimmunoassay (RIA) or immunoenzymatic assay (EIA) methods. CEA was measured by Lepetit Lysophase RIA (Milano, Italy) and successively by Sorin Biomedica (Saluggia, Italy) commercial kits; serum levels $>7\ \mathrm{ng\ ml^{-1}}$ were considered elevated.

TPA was measured by Sangtec Medical (Bromma, Sweden) commercial kit; serum levels initially $>60\ \mathrm{mU\ ml^{-1}}$ and successively $>85\ \mathrm{mU\ ml^{-1}}$ were considered elevated.

Serum CA15-3 concentrations were determined by IRMA (Cis International) commercial kit and $32\ \mathrm{U\ ml^{-1}}$ was taken as the cut-off level.

The CEA, TPA and CA15-3 within and between assay variations were less than 6% and 9% respectively. When the TPA cut off value was $60\ \mathrm{mU\ ml^{-1}}$ its variation coefficients increased to 10% and 15% respectively.

In the 241 patients without relapse, serum CEA, TPA and CA15-3 determinations were performed 1374, 1350 and 1360 times respectively, while in the 44 patients with metastases they were carried out 282, 283 and 276 times.

*Sensitivity of CA15-3, CEA, TPA in the 'early' diagnosis of breast cancer metastases and in relapsed patients*

During the post-operative follow-up in patients without any clinical sign of relapse and all three tumour markers within the normal range, imaging techniques were performed at regular intervals according to a fixed protocol reported previously (Nicolini *et al.*, 1989).

Moreover, when any patient was suspected of relapse by tumour markers or clinically, radiological examinations were immediately carried out to confirm the suspicion and to define the site of metastases.

Increase of markers were subdivided into: isolated elevated value, constant elevation and progressive increase.

In particular, when an elevated value of one or more tumour markers occurred, another sample was taken within 1 month. When the tumour marker's increase decreased to a normal level after a high value, it was considered as isolated elevated value. The tumour marker elevation was considered progressive when a high value increased by $\geqslant 30\%$ in the sample following the elevation. Otherwise, these two high values were regarded as constant elevation.

In the 'early' diagnosis of breast cancer metastases, true positive patients were considered when one or more tumour markers increased simultaneously or preceded the clinical and imaging signs of metastases, and the following sample confirmed the marker increase. As to sensitivity of the 27 patients who relapsed during the follow-up, for CEA and TPA it was evaluated in them all, while for CA15-3 it was evaluated in 24.

Sensitivity of CA15-3, CEA, TPA was also evaluated in 40 relapsed patients which included those where tumour markers had not been the first pathological finding or who had started monitoring with tumour markers after clinical and/or radiological signs of distant metastases.

Among these, we considered true positives those who during a follow-up longer than 1 year and in more than two samples showed high tumour marker levels probably due to metastatic disease. We assumed that as sensitivity in relapsed patients and it allowed us to investigate the frequency of expression of the three tumour markers.

*CA15-3, CEA, TPA specificity in the post-operative follow-up*

In a group of 169 patients without any clinical and radiological signs of relapse and followed-up longer than 24 months (40 ± 8; mean ± s.d.), constant elevation or progressive increase in serum CEA, TPA and CA15-3 levels were considered falsely positive results when not explained by concomitant benign disease. In fact, data from our previous work (Nicolini *et al.*, 1989), showed that an isolated elevated tumour marker level hardly ever suggests breast cancer metastases.

*Evaluation of CA15-3, CEA and TPA sensitivity for monitoring the response to therapy in metastatic patients*

CA15-3, CEA and TPA sensitivity for monitoring the response to therapy was investigated in the group of 40 relapsed patients. In all evaluable patients, clinical and radiological signs were compared with mean serum levels of tumour markers at the relapse, 3 to 6 and 7 to 12 months after the beginning of treatment (D = percentage of difference with mean serum levels at relapse). Only three conditions were considered: progression (slight and strong), remission (partial and complete) and stable disease.

Criteria to define variations of the clinical picture were as follows: slightly progressive disease (appearance or mild worsening of symptoms likely related to the relapse with or without corresponding radiological signs) strongly progressive disease (overall worsening of clinical picture with corresponding radiological signs, i.e. 50% or greater increase in the measurable lesions or appearance of new lesions) partial response (disappearance or mild improvement of symptoms likely releated to the relapse with or without corresponding radiological signs) complete response (overall improvement of clinical picture with corresponding radiological signs, i.e. 50% of greater reduction in the measurable lesions and no appearance or new lesions) stable disease (no variation of symptoms and radiological signs). Patients with progressive disease evaluated in relation to serum CA15-3, CEA and TPA levels were 11, 10, 20, those with remission 6, 5, 8 and those with stable disease were 4, 3, 8 respectively.

### Results

*Sensitivity of CA15-3, CEA, TPA in the 'early' diagnosis of breast cancer metastases and in relapsed patients*

In 21 breast cancer patients who relapsed during the follow-up serum levels increase of one or more tumour markers preceded the clinical and/or radiological signs of distant metastases. The highest sensitivity was shown by TPA alone and the CA15-3-CEA-TPA combination (Table I).

In particular, in 11 patients, high serum CA15-3 values were the first pathological finding of relapse. In nine of them, the time elapsed between CA15-3 increase and the clinical

**Table I** Sensitivity, specificity and positive predictive value of CA15-3, CEA, TPA in the 'early' diagnosis of breast cancer metastases

| *Markers* | *Sensitivity %* | *Specificity %* | *Positive predictive value %* |
|---|---|---|---|
| CA15-3 | 46 | 98 | 78 |
| CEA | 7 | 99 | 67 |
| TPA | 63 | 98 | 85 |
| CA15-3 + CEA | 50 | 98 | 75 |
| CA15-3 + TPA | 83 | 96 | 77 |
| CEA + TPA | 70 | 98 | 86 |
| CA15-3 + CEA + TPA | 87 | 96 | 78 |

and/or radiological signs of distant metastases was 2.7 ± 2.6 months (mean ± s.d.), while in the two others receiving tamoxifen adjuvant treatment it was 18 and 24 months respectively. In 17 patients, high serum TPA values were the first sign of distant metastases. In 15 of them, the time elapsed between TPA increase and the certain signs of relapse was 3.4 ± 2.7 months (mean ± s.d.) while in the two remaining patients receiving tamoxifen it was 18 and 20 months respectively.

With regard to CEA, high serum values occurred contemporaneously or preceded 3 months respectively the clinical and radiological signs of distant metastases in only two patients. High serum CA15-3, CEA and TPA values were found in 21 (52%), 18 (45%) and 36 (90%) respectively of the 40 relapsed patients evaluated. When three tumour markers were considered in combination, the sensitivity of CA15-3-CEA-TPA association was 95% that of CEA-TPA and CA15-3-TPA combinations was 92%, whilst CA15-3-CEA association showed only a 75% sensitivity.

*Unspecific increase and false positives of CA15-3, CEA, TPA*

The kind of increase and the reasons probably responsible for the tumour marker increase in 169 non-relapsed breast cancer patients with prolonged follow-up, are shown in Table II.

When all increments were taken into account, isolated elevated values of CA15-3, CEA and TPA occurred 18, 16, 109 times in 16 (9%), 14 (8%) and 77 (45%) patients respectively. Constant elevation was found 13, 7, 69 times in seven (4%), four (2.3%), 32 (19%) patients while progressive increase 1, 0, 20 times in one (0.6%), 0, 14 (8%) subjects. Nevertheless, the false positives, that is patients with constant and/or progressive increase of tumour marker not explained by concomitant benign pathology, were four (2.3%), one (0.6%) and three (1.7%) for CA15-3, CEA and TPA respectively. When they were considered in association, CEA-TPA and CA15-3-CEA specificity was 98% while that of CA15-3-TPA and CA15-3-CEA-TPA combinations decreased to 96% (Table I).

*CA15-3 increment before relapse and during treatment of metastatic patients. Site of metastases in the true positives*

Shortly before the appearance of distant metastases in all 12 evaluated patients, constant elevation and/or progressive increase in serum CA15-3 values preceded clinical and/or radiological signs of relapse; in three (25%) of them there was concomitant benign pathology able to explain the marker increase. The only patient who showed also an isolated elevated value was on adjuvant tamoxifen treatment and without benign disease.

During treatment of metastases in all 18 evaluated patients, constant elevation and/or progressive increase occurred. In five (28%) of these 18 patients there were concomitant reasons known to increase the marker's level. In two (11%) patients showing also an isolated elevated value no concomitant benign pathology was found.

Table III shows the site of metastases in patients with elevated values of CA15-3, CEA and TPA. Bone, lung and liver involvement occurred in 16 (76%), six (28%) and three (14%) respectively in patients with an elevated CA15-3, in 12 (67%), five (28%) and four (22%) for CEA, in 27 (75%), ten (28%) and seven (19%) for TPA.

*Evaluation of CA15-3, CEA and TPA sensitivity to monitor the response to therapy in metastatic patients*

In the patients with progressive disease the mean serum CA15-3 ($\mu$ ml$^{-1}$) values were 55.5, 98 and 157 at relapse, 3 to 6 and 7 to 12 months after beginning of therapy respectively. At the same intervals in patients with remission and stable disease they were 139, 100, 85.5 and 37.5, 47, 46 respectively. As regards the mean serum CEA (ng ml$^{-1}$) levels in progressive disease they were 10.3, 28.7 and 38.3 at relapse and the

**Table II** Concomitant benign pathology responsible for three tumour marker's increase in 169 non relapsed breast cancer patients with prolonged follow-up

| Reasons probably responsible for tumour marker's increase | TPA IEV Times | TPA IEV Pts No | TPA IEV Pts % | TPA CE Times | TPA CE Pts No | TPA CE Pts % | TPA PI Times | TPA PI Pts No | TPA PI Pts % | CEA IEV Times | CEA IEV Pts No | CEA IEV Pts % | CEA CE Times | CEA CE Pts No | CEA CE Pts % | CEA PI Times | CEA PI Pts No | CEA PI Pts % | CA15-3 IEV Times | CA15-3 IEV Pts No | CA15-3 IEV Pts % | CA15-3 CE Times | CA15-3 CE Pts No | CA15-3 CE Pts % | CA15-3 PI Times | CA15-3 PI Pts No | CA15-3 PI Pts % |
|---|---|---|---|---|---|---|---|---|---|---|---|---|---|---|---|---|---|---|---|---|---|---|---|---|---|---|---|
| Transient liver failure | 11 | 9 | 5 | 4 | 3 | 1.7 | 4 | 3 | 1.7 | 0 | 0 | 0 | 0 | 0 | 0 | 0 | 0 | 0 | 2 | 2 | 1 | 0 | 0 | 0 | 0 | 0 | 0 |
| Chronic liver failure | 6 | 5 | 3 | 34 | 11 | 6.5 | 12 | 8 | 4.7 | 1 | 1 | 0.6 | 1 | 1 | 0.6 | 0 | 0 | 0 | 5 | 4 | 2.3 | 3 | 1 | 0.6 | 1 | 1 | 0.6 |
| Diabetes and/or hepatic steatosis | 30 | 22 | 13 | 20 | 9 | 5 | 3 | 2 | 1 | 3 | 3 | 1.7 | 3 | 1 | 0.6 | 0 | 0 | 0 | 3 | 3 | 1.7 | 6 | 2 | 1 | 0 | 0 | 0 |
| Transient hyperglycemia | 5 | 3 | 1.7 | 1 | 1 | 0.6 | 0 | 0 | 0 | 0 | 0 | 0 | 0 | 0 | 0 | 0 | 0 | 0 | 0 | 0 | 0 | 0 | 0 | 0 | 0 | 0 | 0 |
| Smoking | 0 | 0 | 0 | 0 | 0 | 0 | 0 | 0 | 0 | 4 | 3 | 1.7 | 2 | 1 | 0.6 | 0 | 0 | 0 | 0 | 0 | 0 | 0 | 0 | 0 | 0 | 0 | 0 |
| Acute joint inflammation | 5 | 4 | 2.3 | 1 | 1 | 0.6 | 0 | 0 | 0 | 0 | 0 | 0 | 0 | 0 | 0 | 0 | 0 | 0 | 0 | 0 | 0 | 0 | 0 | 0 | 0 | 0 | 0 |
| Acute upper airways inflammation | 4 | 3 | 1.7 | 1 | 1 | 0.6 | 0 | 0 | 0 | 0 | 0 | 0 | 0 | 0 | 0 | 0 | 0 | 0 | 0 | 0 | 0 | 0 | 0 | 0 | 0 | 0 | 0 |
| Miscellanea | 8 | 6 | 3.5 | 5 | 3 | 1.7 | 1 | 1 | 0.6 | 0 | 0 | 0 | 0 | 0 | 0 | 0 | 0 | 0 | 1 | 1 | 0.6 | 0 | 0 | 0 | 0 | 0 | 0 |
| Unknown | 40 | 25 | 15 | 3 | 3 | 1.7 | 0 | 0 | 0 | 8 | 7 | 4 | 1 | 1 | 0.6 | 0 | 0 | 0 | 7 | 6 | 3.5 | 4 | 4 | 2.3 | 0 | 0 | 0 |
| Total | 109 | 77 | 45 | 69 | 32 | 19 | 20 | 14 | 8 | 16 | 14 | 8 | 7 | 4 | 2.3 | 0 | 0 | 0 | 18 | 16 | 9 | 13 | 7 | 4 | 1 | 1 | 0.6 |

Pts = patients; IEV = isolated elevated value; CE = constant elevation; PI = progressive increase.

**Table III** Site of metastases in patients with elevated serum CA15-3, CEA and TPA values

| *Marker* | *Pts evaluated n* | *B* | *L* | *Li* | *B-L* | *L-Li* | *B-Li* | *B-L-Li* | *P* | *Br* | *S* |
|---|---|---|---|---|---|---|---|---|---|---|---|
| | | *Site of repetitions* | | | | | | | | | |
| CA15-3 | 21 | 12 | 3 | 1 | 2 | – | 1 | 1 | – | – | 1 |
| CEA | 18 | 9 | 2 | 1 | 1 | 1 | 1 | 1 | – | – | 2 |
| TPA | 36 | 19 | 4 | 1 | 3 | 1 | 3 | 2 | 1 | 1 | 1 |

Pts = patients; B = bone; L = lung; Li = liver; P = pleura; Br = brain; S = skin.

following intervals while in patients with remission and stable disease they were 13.6, 11, 11 and 7.8, 8.1, 10.2 respectively. The mean serum TPA (mU ml$^{-1}$) values in patients with progression were 170, 277 and 491 at the fixed intervals, while in those with remission and stable disease they were 251, 152, 59.1 and 113, 105 and 111 respectively. Therefore in the progressive disease serum increase of three markers occurred 3 to 6 (D = + 76.5%, + 179% and + 63% respectively) and still more (D = + 183%, + 272% and + 188% respectively) 7 to 12 months after relapse. In patients with remission, all three markers decreased 3 to 6 months after the appearance of metastases (D = – 28%, – 19% and – 39% respectively). At the following interval CA15-3 and TPA values further decreased (D = – 38% and – 76% respectively) while mean serum CEA levels did not change.

In patients with stable disease, CA15-3 and CEA values both at 3 to 6 (D = + 25.6% and + 3.8% respectively) and 7 to 12 months intervals (D = + 23% and + 30% respectively) were higher than at relapse while at the same intervals serum TPA levels slightly decreased (D = – 7% and – 1.7% respectively).

### Discussion

In the 'early' diagnosis of breast cancer relapses, CA15-3 showed a sensitivity much higher than CEA and lower than TPA (Table I). The mean interval between serum CA15-3 increase and the appearance of signs of distant metastases was similar to those of TPA and CEA (2.7 ± 2.6 *vs* 3.4 ± 2 and 1.5 ± 2 months) respectively. This last result shows that in most breast cancer patients, serum CA15-3 levels as with CEA and TPA increase a few months before clinical and/or radiological signs of distant metastases. Nevertheless, the much longer interval (18 to 24 months) found for CA15-3 or TPA in four patients receiving tamoxifen confirms earlier data (Nicolini *et al*., 1989; Fisher *et al*., 1981; Henderson *et al*., 1988; Palshof *et al*., 1980), in which in hormone-responsive patients, 'early' or adjuvant treatment with tamoxifen significantly delays progression of disease and prolongs the interval from tumour marker increase to the clinical and/or radiological signs of relapse.

In nonrelapsed patients, isolated elevated value of all three tumour markers occurred more often than constant elevation and this type of increment more than progressive increase (Table II). Moreover, most of these tumour marker increases were concomitant with benign disease, in particular consistent with previous data (Nicolini *et al*., 1989; Colomer *et al*., 1986; Ruibal *et al*., 1986*a*, *b*), hepatic diseases (transient or chronic liver failure, diabetes and/or hepatic steatosis) seem to be the most commonly involved. Thus a very high specificity ranging from 96 to 99% was obtained both when three tumour markers were considered alone and in association (Table I).

In all metastatic patients with high serum CA15-3 values before and during treatment constant elevation and/or progressive increase in this tumour marker were found and only in three of them also isolated elevated value occurred. Therefore it can be inferred that, as we reported for CEA and TPA (Nicolini *et al*., 1989) mainly in patients without concomitant benign disease, CA15-3 constant elevation or progressive increase unlike isolated elevated value strongly suggest breast cancer relapse.

In relapsed patients, TPA showed the highest sensitivity (90%) while only in about half of them high CA15-3 and CEA levels occurred. This indicates that most breast cancer metastases produce TPA and only about half of them CA15-3 and CEA.

In these patients, no organ specificity was shown either by CA15-3 or by CEA and TPA. In fact, when all sites of metastases were considered, no significant difference was found among the percentages of patients with high CA15-3 values and those with elevated CEA or TPA concentrations, with regard to the frequency of the main target organs involved (bone, lung, liver) (Table III). On the other hand, each of the three tumour markers showed a higher percentage in patients with bone metastases than that of patients with lung involvement and the percentage was even less in patients with liver metastases (Table III).

Serum TPA levels reflected the variations of metastatic disease better than CA15-3 and CEA. In fact, when serum values of the three markers were compared with the clinical and/or radiological variations, in progressive disease at both the evaluated intervals, the percentage of mean serum CEA increase was higher than that of CA15-3 and TPA. Nevertheless, in patients with remission and stable disease mean serum CEA values showed the lowest decrease (D = – 19%) and the highest difference (D = + 30%) respectively. Better correspondence was given by CA15-3 and even more by TPA.

These findings are consistent with previous studies at least as regards TPA and CEA (Luthgens & Schlegel, 1981; Skryten *et al*., 1981; Biorklund, 1983; Quayle, 1982) and suggest that TPA is related to tumoral proliferation while CA15-3 and CEA are tumour mass related antigens. In conclusion, data from this study indicate that in the post-operative follow-up of breast cancer patients, TPA is the most useful of the three tumour markers and CA15-3 TPA is the most suitable combination. In spite of a slight decrease of positive predictive value in respect of the CEA-TPA combination (77% *vs* 86%), CA15-3 more significantly increased TPA sensitivity in the 'early' diagnosis of breast cancer relapses (Table I). Moreover, monitoring the response to therapy serum CA15-3 levels showed better correspondence with the clinical and radiological variations than the CEA levels. Contemporaneous measurement of serum CEA levels only slightly increases sensitivity and positive predictive value of CA15-3-TPA association (Table I).

### References

BIORKLUND, B. (1983). Tumor products reflecting growth activity. In *Cancer Treatment: End Point Evaluation*, Stoll, B.A. (ed.), p. 251. John Wiley: New York.

COLOMER, R., RUIBAL, A., NAVARRO, M., ENCABO, G., SOLE, L.A. & SALVADOR, L. (1986). Circulating CA15-3 levels in breast cancer. Our present experience. *Int. J. Biol. Markers*, **1**, 89.

FISHER, B., REDMOND, C., BROWN, A., WOLMARK, N., WITTLIFF, J. & FISHER, E.R. (1981). Treatment of primary breast cancer with chemotherapy and tamoxifen. *N. Engl. J. M*., **305**, 1.

GION, M., MIONE, R., DITTADI, R., FASAN, S., PALLINI, A. & BRUSCAGLIN, G. (1986). Evaluation of CA15-3 serum levels in breast cancer patients. *J. Nucl. Med & All. Sci*., **30**, 29.

GORSKY, Y., VANKY, F. & SULITZEANU, D. (1976). Isolation from patients with breast cancer of antibodies specific for antigens associated with breast cancer and other malignant diseases. *Proc. Natl Acad. Sci. USA*, **73**, 2101.

HAAGENSEN, D.E. Jr. (1982). Tumor markers for breast carcinoma. *Clin. Lab. Med.*, **2**, 543.

HENDERSON, I.C., MOURIDSEN, H., ABE, O. & 76 others (1988). Effects of adjuvant tamoxifen and of cytotoxic therapy on mortality in early breast cancer. An overview of 61 randomized trials among 28,896 women. Early breast cancer trialists' collaborative group. *N. Engl. J. Med.*, **319**, 1681.

HILKENS, J., KROEZEN, V., BONFRER, J.M.G., BRUNING, P.F., HILGHERS, J. VAN EIJKEREN, A. (1984). A sandwich-radioimmunoassay for a new antigen (MAM-6) present in the sera of the patients with metastatized carcinomas. In *Protides of the Biological Fluids* 32nd, Peeters, H. (ed.), p. 651. Pergamon Press: Oxford.

HUMPHREY, L.J., ESTES, N.C., MORSE, P.A. Jr., JEWELL, W.R., BOUDET, R.A. & HUDSON, N.J.K. (1974). Serum antibody in patients with mammary disease. *Cancer*, **34**, 1516.

LUTHGENS, M. & SCHLEGEL, G. (1981). Verlaufskontrolle mit tissue polypeptide antigen und carcinoembryonalem antigen in der radioonkologischen nachsorge und therapie. *Tumor Diagnostik*, **2**, 179.

MAIDMENT, B.W. Jr., PAPSIDERO, L.D., NEMOTO, T. & MING CHU, T. (1981). Recovery of immunologically reactive antibodies and antigens from breast cancer immuno-complexes by preparative isoelectric focusing. *Cancer Res.*, **41**, 795.

NICOLINI, A., CARPI, A., DI MARCO, G., GIULIANI, L., GIORDANI, R. & PALLA, S. (1989). A rational post-operative follow-up with carcinoembryonic antigen, tissue polypeptide antigen and urinary hydroxyproline in breast cancer patients. *Cancer*, **63**, 2037.

OMAR, Y.T., BEHBEHANI, A.E., AL-NAQEEB, N. & 5 others (1988). Carcinoembryonic antigen and breast carcinoma antigen (CA15-3) in preoperative staging and post-operative monitoring of patients with carcinoma of the breast. *Int. J. Biol. Markers*, **3**, 165.

PALSHOF, T., MOURIDSEN, H.T. & DAEHNFELDT, J.L. (1980). Adjuvant endocrine therapy of primary operable breast cancer. Report on the Copenhagen breast cancer trials. *Eur. J. Cancer*, Suppl. **1**, 183.

QUAYLE, J.B. (1982). Ability of CEA blood levels to reflect tumour burden. A study in a human xenograft model. *Br. J. Cancer*, **46**, 220.

RUIBAL, A., GENOLLA, J., ROSELL, M., GRIS, M.J. & COLOMER, R. (1986*a*). Serum CA15-3 levels in patients with non-tumoral diseases and establishment of a threshold for tumoral activity-results in 1219 patients. *Int. J. Biol. Markers*, **1**, 159.

RUIBAL, A., ENCABO, G., GENOLLA, V., GUARGA, A., URRUTIA, A. & COLOMER, R. (1986*b*). Serum CA15-3 levels in patients with general pathology and malignant diseases (excluding breast cancer). *Bull. Cancer (Paris)*, **73**, 94.

SCHMIDT-RHODE, P., SCHULZ, K.D., STURM, G., RAAB-FRICK, A. & PRINZ, H. (1987). CA15-3 as a tumor marker in breast cancer. *Int. J. Biol. Markers*, **2**, 135.

SKRYTEN, A.F., UNSGAARD, B., BJORKLUND, B. & EKLUND, G. (1981). Serum TPA related to activity in a wide spectrum of cancer conditions. *Tumor Diagnostik*, **3**, 117.